# To Be or Not to Be a Flatworm: The Acoel Controversy

**DOI:** 10.1371/journal.pone.0005502

**Published:** 2009-05-11

**Authors:** Bernhard Egger, Dirk Steinke, Hiroshi Tarui, Katrien De Mulder, Detlev Arendt, Gaëtan Borgonie, Noriko Funayama, Robert Gschwentner, Volker Hartenstein, Bert Hobmayer, Matthew Hooge, Martina Hrouda, Sachiko Ishida, Chiyoko Kobayashi, Georg Kuales, Osamu Nishimura, Daniela Pfister, Reinhard Rieger, Willi Salvenmoser, Julian Smith, Ulrich Technau, Seth Tyler, Kiyokazu Agata, Walter Salzburger, Peter Ladurner

**Affiliations:** 1 Institute of Zoology and Center for Molecular Biosciences, University of Innsbruck, Innsbruck, Austria; 2 Biodiversity Institute of Ontario, University of Guelph, Guelph, Ontario, Canada; 3 Evolutionary Regeneration Group, Center for Developmental Biology, RIKEN Kobe, Kobe, Japan; 4 Department of Biology, Nematology Section, University of Ghent, Ghent, Belgium; 5 Developmental Biology Programme, EMBL, Heidelberg, Germany; 6 Department of Molecular, Cell and Developmental Biology, University of California Los Angeles, Los Angeles, California, United States of America; 7 School of Biology and Ecology, University of Maine, Orono, Maine, United States of America; 8 Department of Biophysics, Graduate School of Science, Kyoto University, Kyoto, Japan; 9 Department of Biofunctional Science, Faculty of Agriculture and Life Sciences, Hirosaki University, Hirosaki, Japan; 10 Division of Integrative Cell Biology, Institute of Molecular Embryology and Genetics, Kumamoto University, Kumamoto, Japan; 11 Department of Biology, Winthrop University, Rock Hill, South Carolina, United States of America; 12 Department for Molecular Evolution and Development, Centre for Organismal Systems Biology, Faculty of Life Sciences, University of Vienna, Vienna, Austria; 13 Zoological Institute, University of Basel, Basel, Switzerland; University of Texas Arlington, United States of America

## Abstract

Since first described, acoels were considered members of the flatworms (Platyhelminthes). However, no clear synapomorphies among the three large flatworm taxa - the Catenulida, the Acoelomorpha and the Rhabditophora - have been characterized to date. Molecular phylogenies, on the other hand, commonly positioned acoels separate from other flatworms. Accordingly, our own multi-locus phylogenetic analysis using 43 genes and 23 animal species places the acoel flatworm *Isodiametra pulchra* at the base of all Bilateria, distant from other flatworms. By contrast, novel data on the distribution and proliferation of stem cells and the specific mode of epidermal replacement constitute a strong synapomorphy for the Acoela plus the major group of flatworms, the Rhabditophora. The expression of a *piwi*-like gene not only in gonadal, but also in adult somatic stem cells is another unique feature among bilaterians. These two independent stem-cell-related characters put the Acoela into the Platyhelminthes-Lophotrochozoa clade and account for the most parsimonious evolutionary explanation of epidermal cell renewal in the Bilateria. Most available multigene analyses produce conflicting results regarding the position of the acoels in the tree of life. Given these phylogenomic conflicts and the contradiction of developmental and morphological data with phylogenomic results, the monophyly of the phylum Platyhelminthes and the position of the Acoela remain unresolved. By these data, both the inclusion of Acoela within Platyhelminthes, and their separation from flatworms as basal bilaterians are well-supported alternatives.

## Introduction

Flatworms (phylum Platyhelminthes) have long been considered the most basal bilaterians, and they have served as models for the bilaterian ancestor in a variety of phylogenetic hypotheses. Generally, morphological data place the Acoela within the Platyhelminthes based on a combination of weak characters: an acoelomate body structure, a densely multiciliated monolayered epidermis leading to a common habitus, a frontal organ, neoblasts, hermaphroditic reproduction with similar reproductive-organ morphology, biflagellate sperms with inverted axonemes (in acoels and rhabditophorans except macrostomorphans), and lack of hindgut and anus [1]–[3]. But already one year after the most comprehensive morphological phylogenetic system of the Platyhelminthes was published [2], the monophyly of the group was questioned on the basis of the ambiguity of the uniting characters and because of the absence of outgroups in the assessment of the suitability of these characters as apomorphies [4]. In molecular phylogenetic analyses the position of acoels remained unresolved as well: Acoels are placed well outside the Platyhelminthes as a sister group to the other bilaterians based on data from a single gene or a few loci only, such as 18S and 28S rDNA, Hox and ParaHox genes, myosin II, or microRNA [5]–[11]. In multigene analyses, acoels appear within the Lophotrochozoa [12], or they are associated with deuterostomes [13], or they are basal bilaterians [14]. Because morphological characters are incongruent with the various molecular phylogenetic hypotheses, the placement of Acoela remains controversial; previous attempts to subsume molecular and morphological data proved unsatisfactory (reviewed in [9]).

We have succeeded in finding two strong synapomorphies between acoel and rhabditophoran flatworms. The stem cell system and the particular mode of replacing epidermal cells represent unique features shared by both acoel and rhabditophoran flatworms, but not by any other bilaterian lineage. At the same time, our phylogenomic data support a separation of acoels from rhabditophoran flatworms.

## Results and Discussion

### Phylogenomics place the Acoela at the base of the Bilateria

**Figure 1:**
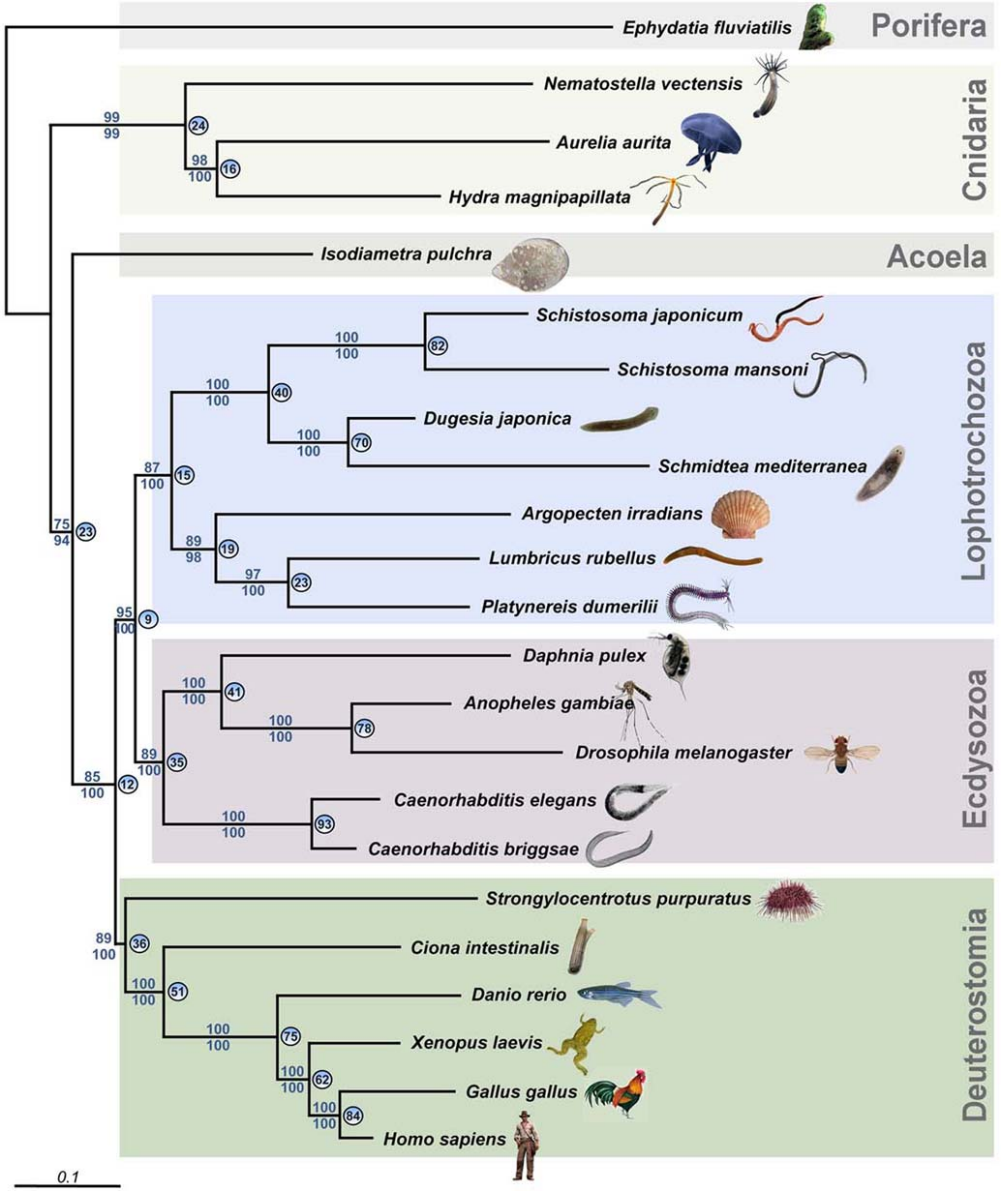
Phylogenetic analysis of 23 animal species using partial sequences of 43 genes. The acoel *Isodiametra pulchra* appears as a sister group of the rest of the bilaterians, and not as a member of the Platyhelminthes. Numbers above nodes refer to the maximum likelihood boostraps. Values below nodes represent bootstrap support under CAT. Circled numbers indicate the percentage of individual-loci trees that supported the respective node in the maximum-likelihood analyses of each data-set separately.

Here, we provide new molecular and developmental data having a bearing on the flatworm controversy. We produced ESTs from several species: the cnidarian *Aurelia aurita* and *Nematostella vectensis*, the sponge *Ephydatia fluviatilis*, the acoel *Isodiametra pulchra*, the flatworm *Macrostomum lignano*, and the annelid *Platynereis dumerilii.* Applying a phylogenomic approach on the basis of 10,218 amino acid positions of the acoel *Isodiametra pulchra*, we first identified a set of open reading frames homologous to sequences we generated from major animal taxa or which were represented in public databases. To avoid the use of paralogs, we limited our selection of genes to e-values ≤10^−50^ in blast searches. We subjected the resulting multi-locus datasets to phylogenetic analyses using Maximum likelihood and Bayesian inference and analyzed two datasets consisting of 23 species represented by 43 loci (Fig. 1) or 24 species represented by 32 loci (Fig. S1, same dataset, but including the rhabditophoran *Macrostomum lignano*), representing diverse animal phyla and including partial sequence data (see Supplementary Material and Tables S1 and S2).

In contrast to two previous multi-locus approaches [12]–[13], our new molecular phylogeny puts the acoels basal to all other bilaterians (Fig. 1, Fig. S1). The remaining flatworms consistently appear at the base of the Lophotrochozoa and close to coelomate spiralian phyla such as Mollusca and Annelida (cf., e.g. [13]). Our multi-locus phylogenetic analysis suggests, as others have, a separation of acoels from rhabditophoran flatworms and a sister-group relationship of acoels to the remaining bilaterians. Apart from the position of the acoels, the overall topology of the tree inferred by our approach is congruent with the current view of animal evolution [12]–[15].

### The unique stem cell system unites acoel and rhabditophoran flatworms

**Figure 2:**
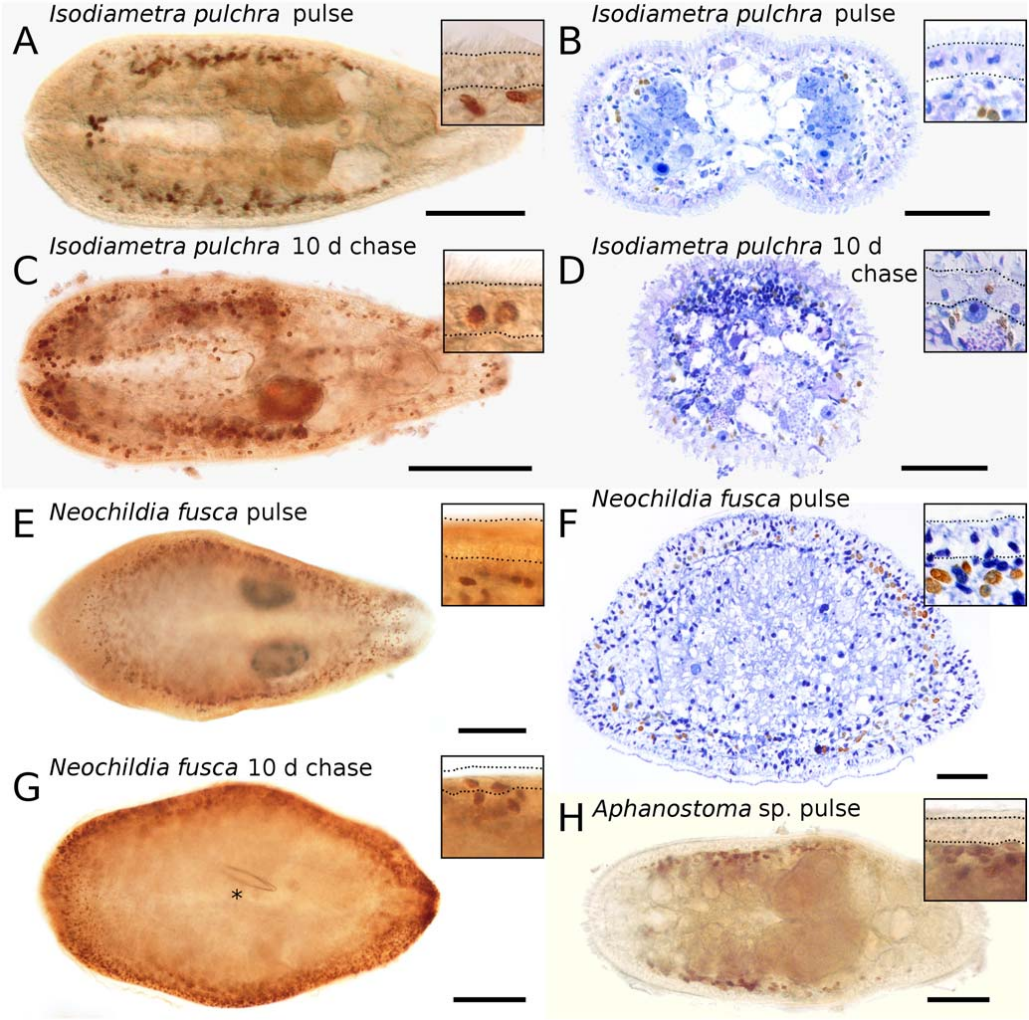
Cell proliferation and cell migration in acoel flatworms. Localization of BrdU-containing cells in the acoels *Isodiametra pulchra*, *Neochildia fusca* and *Aphanostoma* sp. after a short BrdU pulse (A–B, E–F, H) and 10 days chase (C–D, G). (A, C, E, G–H) wholemounts of adult animals, (B, D, F) semithin cross sections. Insets: details of the epidermis, encompassed by dotted lines. Anterior to the left. (A–B, E–F, H) Note the lack of S-phase cells (brown nuclei) in the epidermis after 30 min BrdU pulse. (C–D, G) BrdU-labeled cells (brown nuclei) migrated from the mesodermal space to the epidermis and differentiated into epidermal cells during the 10 days chase period. Asterisk denotes diatom in digestive parenchyma. Scale bar is 50 µm for (B, D, F, H), 100 µm for (A, C), and 200 µm for (E, G).

**Figure 3:**
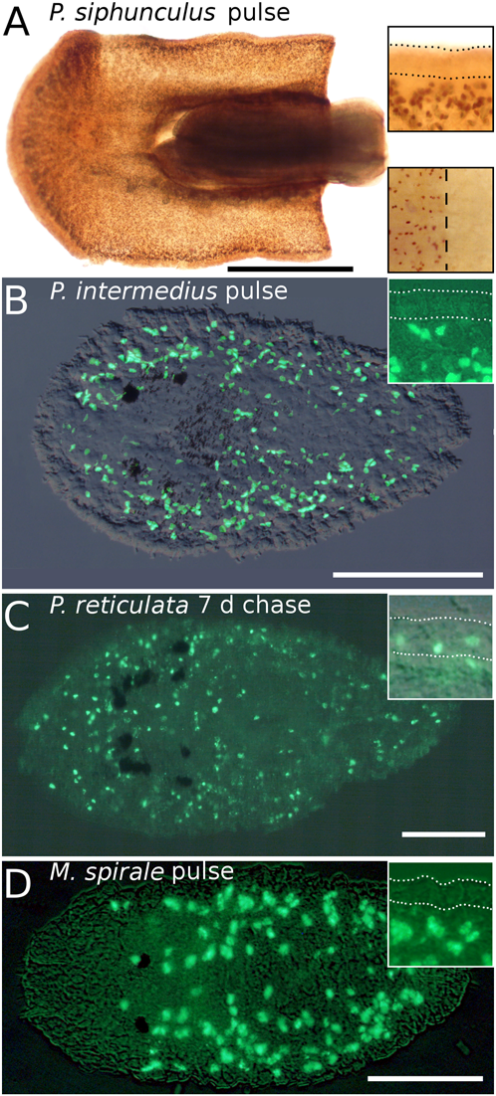
Cell proliferation and cell migration in rhabditophoran flatworms. Localization of BrdU-containing cells in the rhabditophorans *Prosthiostomum siphunculus*, *Pseudostylochus intermedius*, *Planocera reticulata* (A–C, Polycladida) and *Macrostomum spirale* (D, Macrostomorpha). (A) Anterior part of an adult, (B–D) juveniles. Insets: details of the epidermis, encompassed by dotted lines. Anterior to the left. (A–B, D) Note the lack of S-phase cells (brown or green nuclei) in the epidermis after 30 min or 12 h (in B) BrdU pulse. (A) Lower inset shows the protruding pharynx also lacking proliferating cells. (C) BrdU-labeled cells (green nuclei) migrated from the mesodermal space to the epidermis and differentiated into epidermal cells during the 7 days chase period. Scale bar is 50 µm for (D), 100 µm for (B), 400 µm for (C) and 1 mm for (A).

**Figure 4:**
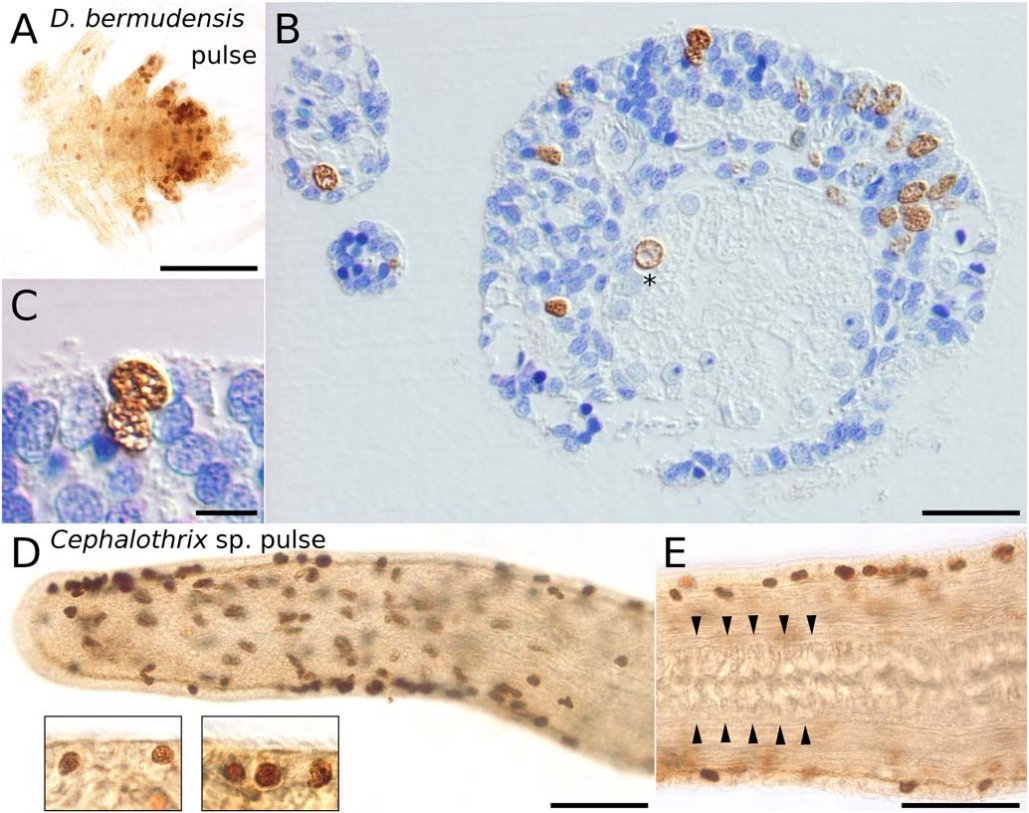
Cell proliferation in other spiralians: an annelid and a nemertean. Localization of BrdU-containing cells in the annelid *Dorvillea bermudensis* (A–C) and the nemertean *Cephalothrix* sp. (D–E) after 30 min incubation of BrdU. (A) Wholemount of the posterior segments of *D. bermudensis*. (B) Semithin cross section through midbody and parapodia of animal shown in (A). Labeled cells are located in the epidermis, the mesodermal space, and the gastrodermis (indicated by asterisk). (C) Magnified view of labeled epidermal cells shown in (B). (D) Anterior end of *Cephalothrix* sp. Labeled cells in the epidermis are separated from muscular layers and the cutis by a light-brown basal matrix. Labeled cells are also present in the mesodermal space. Inset shows details of labeled epidermal cells. (E) More posterior part of the animal than (D) with labeled cells in the epidermis. Arrowheads denote the proboscis. Scale bar is 100 µm for (A), 20 µm for (B), 5 µm for (C), and 50 µm for (D–E).

**Figure 5:**
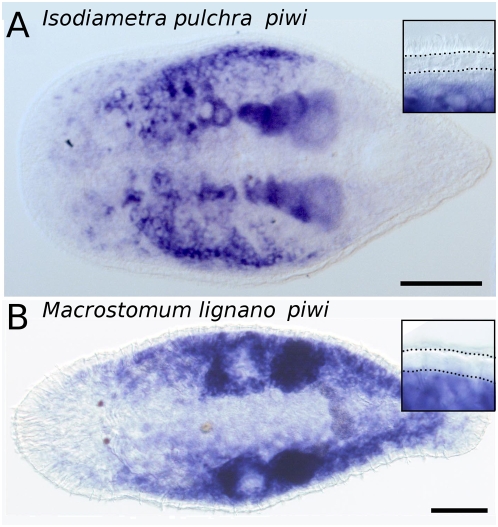
*Piwi*-like gene expression in an acoel and a rhabditophoran flatworm. *In situ* hybridizations of adult animals. (A) *Isodiametra pulchra* (Acoela), (B) *Macrostomum lignano* (Rhabditophora). Expression of *piwi*-like genes in the germ line and somatic stem cells. Note the lack of *Ipiwi1* (A) and *Macpiwi* (B) expression in the epidermis. Insets: details of the epidermis, encompassed by dotted lines. Accession numbers: *Ipiwi1* AM942741, *Macpiwi* AM942740. Scale bar is 100 µm.

For the analysis of novel developmental data, we focused on the extraordinary stem cell system of flatworms. We mapped the distribution of S-phase stem cells and epidermal replacement in acoel flatworms using three species from two families (Fig. 2), and in rhabditophoran flatworms including four species from two orders and four different families (Fig. 3). We also included, for comparative reasons, the annelid *Dorvillea bermudensis* and the nemertean *Cephalothrix* sp. in the analysis of the distribution of S-phase stem cells (Fig. 4). Additionally, we performed *in situ* hybridization experiments with the stem-cell-specific marker *piwi* in the acoel flatworm *I. pulchra* and the rhabditophoran flatworm *M. lignano* (Fig. 5).

Our experiments clearly demonstrate that, in acoels, epidermal cells are exclusively renewed from mesodermally located stem cells (Fig. 2). The very same mode of epidermal cell renewal and the absence of proliferating cells in the epidermis characterizes rhabditophoran taxa such as macrostomorphans [16] (Fig. 3), polyclads [17] (Fig. 3), triclads [18], rhabdocoels [19] and parasitic platyhelminths [20]. In contrast, proliferating cells in the epidermis occur in all other lophotrochozoans investigated, including annelids [21] (Fig. 4A–C), nemertines (Fig. 4D–E), and molluscs [22]. Thus, it is the nature of epidermal replacement - through stem cells originating from the mesodermal space rather than the epidermis itself - that sets acoels and rhabditophorans apart from other bilaterian taxa.

Gene expression patterns of the stem-cell marker *piwi* further substantiate the acoel-rhabditophoran grouping. Within the Bilateria, *piwi*-like genes are highly evolutionarily conserved, and expression is largely restricted to the germline, where it plays an important role in germ-cell development and maintenance, in meiosis, as well as in the regulation of retrotransposons [23]–[24]. In most animals studied so far, *piwi* RNA interference results in sterility. However, in triclads, as well as in *M. lignano* and *I. pulchra*, *piwi*-like gene expression is extended to a subpopulation of somatic stem cells [25]–[28]. Downregulation of *piwi*-like genes in flatworms results in loss of tissue homeostasis and regeneration capacity, which finally leads to death [25]–[26]. These observations suggest a crucial role of *piwi*-like genes in somatic stem-cell maintenance in flatworms. Also, in the acoel *I. pulchra*, we were able to show the extended *Ipiwi1* expression in a subpopulation of somatic stem cells, suggesting a similar regulation of both acoel and rhabditophoran stem-cell systems. Furthermore, consistent with the conspicuous absence of proliferating cells in the epidermis, the flatworm stem cell marker *piwi* is not expressed in the epidermal layer [25], [26]. Accordingly, we demonstrate that in the rhabditophoran *M. lignano*, and in the acoel *I. pulchra*, *piwi*-like genes are expressed in gonads and somatic stem cells, but not in the epidermis (Fig. 5).

### Alternative 1: The stem-cell system is a synapomorphy of Acoela and Rhabditophora

**Figure 6:**
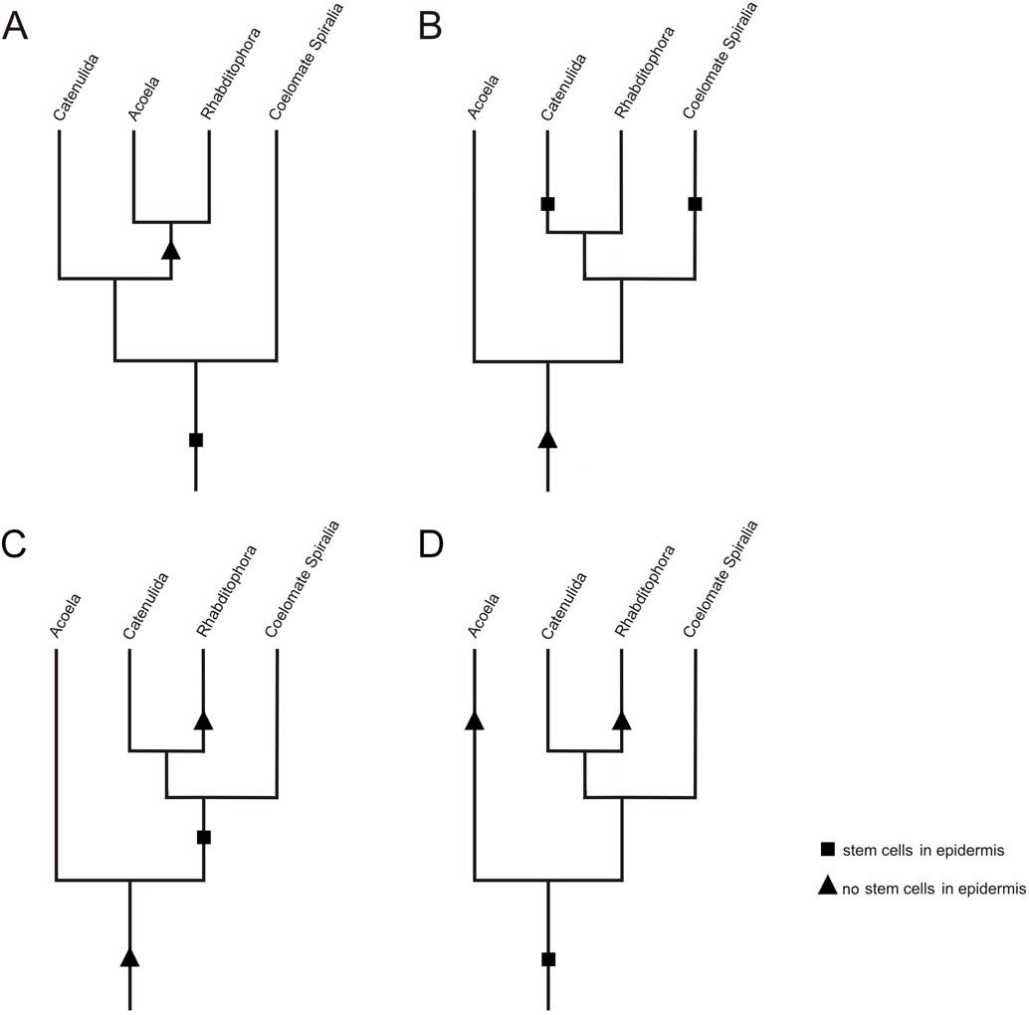
Alternative hypotheses of evolution of epidermal replacement. (A) Alternative 1: The similar stem-cell system between Acoela and Rhabditophora is a synapomorphy. This scenario requires a single loss of epidermal stem cells. Notably, the observation of mitotic figures in the epidermis of catenulids supports a sister group relationship of the Catenulida to the Acoela and Rhabditophora. (B) Alternative 2a: The similar stem-cell system between Acoela and Rhabditophora is a plesiomorphy in both taxa. This requires the independent gain of stem cells in the epidermis by the Catenulida, the coelomate Spiralia and other Bilateria not shown in the diagram. (C) Alternative 2b: The similar stem-cell system between Acoela and Rhabditophora is a plesiomorphy in Acoela and a convergent character in Rhabditophora. This requires the gain of stem cells in the epidermis in the Spiralia and other Bilateria not shown in the diagram. (D) Alternative 3: The similar stem-cell system between Acoela and Rhabditophora is a convergent character that was independently developed in both Acoela and Rhabditophora.

The new developmental data attest to a possible sister-group relationship between Acoela and Rhabditophora (Fig. 6A), thereby contradicting the molecular-phylogeny-based separation of the acoel species from other flatworms. Neoblast stem cells are located in the mesodermal space of acoels [29] (Fig. 2) and rhabditophorans [16], [18], [20] (Fig. 3), but are absent in the epidermis. In a previously studied acoel, *Convolutriloba longifissura*, it is less apparent that proliferating cells are missing in the epidermis, due to insunk epidermal nuclei [29].

Morphological characteristics (i.e. the distribution of stem cells and the peculiar mode of epidermal replacement) and gene-expression patterns of the stem-cell marker *piwi* both confirm the unique mode of epidermal replacement by mesodermally located stem cells in acoels and rhabditophorans. This is the first experimental evidence for complex and robust synapomorphic characters of the Acoela and Rhabditophora. Hence, the inclusion of Acoela in the Platyhelminthes can be seen as the most parsimonious explanation for the presence of epidermal cell renewal from mesodermally located stem cells and for the *piwi*-like gene expression in adult somatic stem cells (Fig. 6A). However, the peculiar mode of epidermal cell renewal exclusively from mesodermally located stem cells may not be a synapomorphy for all traditional taxa of the Platyhelminthes, i.e. the Catenulida, the Acoela, the Nemertodermatida and the Rhabditophora [2]. Stem cells in catenulids have long been recognized as being different from those of other flatworms in that they appear in the epidermis [30], [31]. Preliminary data indicate that in some (but not all) Nemertodermatida proliferating cells occur also in the epidermal layer [32]. To justify a monophyletic Platyhelminthes, this character could be regarded as being secondarily derived in the Nemertodermatida and, probably independently, also in the Catenulida.

Interestingly, two previous multi-gene phylogenies show some support for the grouping of acoels with other flatworms: “the standard WAG model groups together the two long branches of Platyhelminthes and the acoel” [14], and the supplementary figures 2 and 3 in [13] feature trees (calculated with maximum parsimony and the WAG model, respectively), where the acoels appear basal to rhabditophoran flatworms. The authors of these papers reject these phylogenies on the basis that the new CAT model is more likely to avoid analytical errors than the previously widely employed WAG model [13], [14].

A number of phylogenetic studies based on single molecules have suggested a more basal position of Acoela and Nemertodermatida [5]–[11], while the monophyly of the Catenulida and the Rhabditophora has been recently confirmed [33]. These phylogenetic hypotheses are difficult to reconcile with the stem-cell system being a synapomorphy between acoel and rhabditophoran flatworms. Yet, phylogenomic approaches have recently challenged the basal bilaterian position of acoel (and nemertodermatid) flatworms [12]–[13]. Given the current state of knowledge, it is possible that the similar stem-cell systems in Acoela and Rhabditophora are plesiomorphic or convergent characters.

On the other hand, totipotency of mesodermally located neoblasts in adult animals could be a character uniting all Platyhelminthes, but so far has only been experimentally tested in the Rhabditophora [34], [35]. For triclad and macrostomorphan flatworms it has been shown that neoblasts are responsible for growth, tissue maintenance, and regeneration of all tissues, including gonads [16], [36]–[38]. Future studies on the totipotency of stem cells of acoels, nemertodermatids and catenulids will be necessary to confirm or rule out this synapomorphy for all traditional flatworm taxa.

### Alternative 2: The stem-cell system of Acoela and Rhabditophora is a plesiomorphy

If the similarity of the complex stem-cell system shared between acoels and rhabditophorans was to be explained as a plesiomorphy derived from a hypothetical urbilaterian (Fig. 6B), and the acoels are considered as basal bilaterians as supported by our phylogeny (Fig. 1), at least the following groups would have to have lost this particular feature: the Deuterostomia, the Ecdysozoa, and the Eutrochozoa (Annelida and Mollusca) (Fig. 1). Other trees featuring more taxa (e.g. [12]) and where acoels hold a different position imply that the particular mode of epidermal cell renewal would even have been lost several more times.

Diploblasts such as cnidarians lack a mesodermal layer, making a direct comparison with the mesodermally located stem cells in the triploblastic acoel and rhabditophoran flatworms difficult. Moreover, recent phylogenetic studies suggest a placozoan-like ancestor of bilaterians instead of a cnidarian (planula)-like one [39].

Epidermal cells are proliferating at least in some cnidarians, and interstitial cells (i-cells) are found and are proliferating in the epidermis between epidermal cells. This is in stark contrast to the neoblasts of acoel and rhabditophoran flatworms, which are entirely lacking in the epidermis. Still, considering the presence of totipotent i-cells in the cnidarian *Hydractinia* [40], homology of these i-cells and the neoblasts in acoels seems possible, but requires multiple gains of epidermally located stem cells in the Bilateria. In this scenario, the stem-cell system in both acoels and rhabditophorans constitutes a plesiomorphy (Fig. 6B), but the same number of gains of stem cells in the epidermis among major bilaterian taxa is necessary even if the stem-cell system is regarded as a plesiomorphy for acoels, and an apomorphy for rhabditophorans (Fig. 6C).

### Alternative 3: The stem-cell system of Acoela and Rhabditophora is a product of convergent evolution

Regardless of whether the neoblast stem-cell system is a plesiomorphy or an apomorphy for acoels, the assumption of an independent development of a very similar stem-cell system in rhabditophorans indicates a similar need in these two taxa for such a peculiar stem-cell system.

Only for the Neodermata (parasitic rhabditophoran flatworms, including tapeworms and flukes), the lack of stem cells within the epidermis can be seen as a prerequisite for avoiding the host defense mechanisms by shedding the ciliated epidermis. During postembryonic development, these parasitic rhabditophoran flatworms completely replace their primary epidermis with a newly formed syncytial epidermal layer derived from mesodermally located stem cells [41]. Some acoels and rhabditophorans share a thin epidermis and weak basal matrix, which might be related to the loss of an intra-epidermal stem-cell system. Many flatworms also share a similar habitat, the mesopsammon [42], but other representatives of the interstitial fauna, such as annelids and nemerteans do have proliferating stem cells in the epidermis (Fig. 4), showing that the acoel and rhabditophoran stem-cell system is not a necessity to survive in this habitat.

### Conclusion

Considering the obvious conflict between molecular phylogenies and morphological data, the monophyly of the flatworms remains undecided. Although molecular phylogenies show a position of the Acoela separate from the remaining flatworms, the stem-cell system provides two strong synapomorphies for the Rhabditophora and the Acoela: 1) epidermal replacement exclusively through mesodermally located stem cells, and 2) expression of a *piwi*-like gene also in somatic, not only in gonadal stem cells. The alternative would be that the highly similar stem-cell system evolved in parallel in Acoela and Rhabditophora, or is a plesiomorphic feature that was retained.

Recently, the myxozoan worm *Buddenbrockia* has been identified as a member of the Cnidaria by molecular means despite striking morphological dissimilarity [15]. While this conflict between morphological and molecular characters can be readily accounted for by the morphological reductionism resulting from the parasitic lifestyle of *Buddenbrockia*, no such accounting can explain the suite of morphological characters shared among Platyhelminthes [2]. In particular, the special mode of epidermal replacement in acoels and rhabditophorans constitutes an apomorphy supporting a possible sister-group relationship between these taxa. The available multi-locus phylogenies, which largely do not even agree with one another concerning the placement of acoels, cannot resolve the validity of a sister-group relationship between the Acoela and the Rhabditophora. The remaining taxa of the traditional Platyhelminthes, the Catenulida and possibly the Nemertodermatida do not share the peculiar stem cell system of Acoela and Rhabditophora and may lie at the base of the flatworms, may have secondarily evolved proliferating stem cells in the epidermis, or may not be flatworms at all. It appears that until substantial sampling of lower taxa among flatworms is performed, and more studies on stem cells in non-rhabditophoran flatworms are available, none of the competing phylogenetic hypotheses can be favored. Therefore, we concur with Tor Karling [1] that “…the search for sister groups throws a sharp light on our insufficient knowledge of the phylogenetic connections [among] the turbellarian taxa….”

## Materials and Methods

### BrdU labeling

BrdU labeling was performed according to [16] except for using 2.5% glutaraldehyde in 0.1 M cacodylate buffer and 9% sucrose for fixation, a StreptABComplex/HRP Duet kit (DAKO) for secondary antibodies and visualization for precipitation of the BrdU label (brown label). Also, different times of BrdU (12 hours pulse instead of 30 min for juvenile of *Pseudostylochus intermedius*) and protease incubation were used for different species, and treatment with 0.1 M HCl after protease incubation was omitted.

### In situ hybridization

Whole mount in situ hybridization (ISH) on *M. lignano* was carried out as described previously [43]. For *I. pulchra*, the same protocol was used, except for Proteinase K treatment, which was applied for 7 min only. Sense and antisense riboprobes were generated using the DIG RNA labeling KIT SP6/T7 (Roche), following the manufacturer's protocol. During hybridization, riboprobes were used at a final concentration of 0.05 ng/µl. The following primer couples were used for generating *in situ* riboprobe templates: For *Macpiwi*, 5′-TGCTCAAGCTGGTGTTGCTGGTC-3′ and 5′-GTCTTGTTGTTGTGCCGCGTGAG-3′. For *Ipiwi1* 5′-CATGCTGGAGATGGGCAAGATCAC-3′ and 5′-GGTGCCGGAGATTTCATTGCTCTC-3. Partial sequences of *piwi*-like genes were obtained from the *Macrostomum lignano* EST database (Angu7606) (Morris et al. 2006) and unpublished *Isodiametra pulchra* ESTs (Contig 447) (Ladurner and Agata, unpublished). Both gene sequences were submitted to genebank (accession number *Ipiwi1* AM943741; *Macpiwi* AM942740). Detailed information of both genes will be published separately.

### Molecular datasets

We used available EST (expressed sequence tags) and whole genome databases in order to obtain multi-locus data matrices for phylogenetic inferences (see Table S1). The approach of combining dozens of homologous gene fragments for phylogenetic reconstruction has been applied successfully in previous phylogenomic studies and seems particularly useful when, for some taxa of interest, only limited genomic resources are available. One advantage of multi-locus phylogenies over single-locus ones is the increase in robustness, which is essentially due to the much larger number of phylogenetic informative positions. Furthermore, an increase in sequence length generally leads to a smaller variance in evolutionary rates and other parameters in model-based phylogeny-reconstruction methods. Concatenation of (many) single gene-alignments may effectively correct for the erroneous phylogenetic signal contained in single genes, and it has been shown that even genes producing incongruent phylogenies are useful in multi-gene alignments, as they may provide additional information for resolving at least some short branches. On the other hand, the analyses of multi-locus datasets are more challenging. For example, it is often difficult, if not impossible, to assign appropriate model parameters for each partition individually. It has been suggested, though, that the increase in the phylogenetic signal and/or signal/noise-ratio due to concatenation has a much stronger effect on the resulting phylogenies than any bias introduced by averaging over model parameters.

Here, we used as starting point a set of 4,885 ESTs from our *Isodiametra pulchra* cDNA-sequencing project (Ladurner and Agata, unpublished). These sequences were used as query for tblastx homology searches against all other 23 databases (Table S1). For these blast searches, we used an e-value ≤10^−50^ to acquire sequences with a large enough degree of sequence homology to be suitable for phylogeny reconstruction and to avoid the erroneous inclusion of paralogs. We then applied EverEST to assign the “best hit” sequences with respect to the *Isodiametra* homolog from every BLAST search. We ended up with a total of 32 loci that were present in all organisms. When excluding the smallest database, *Macrostomum lignano*, for which only 1,231 cDNA fragments were available, we obtained 43 genes that were unambiguously present in all remaining taxa and conserved enough to allow alignment. Homologous protein sequences were aligned with Clustal X, resulting in two datasets containing 32 and 43 fragments, respectively. GenBank accession numbers of the analyzed sequences are listed in Table S2, the number of amino acid positions used for the phylogenetic analyses are listed in Table S3. Outgroup status was assigned to *Ephydatia fluviatilis* because sponges are a valid sister group to the remaining metazoans; cnidarians are a valid sister group to the bilaterians.

### Phylogenetic analyses

Maximum likelihood and Bayesian analyses were performed with both datasets and with single loci as well as multi-gene alignments including all sequence fragments. The total length of the concatenated dataset including *Macrostomum lignano* was 6,718 amino acid positions (32 loci), while the concatenated dataset without *Macrostomum lignano* (43 loci) had 10,218 amino acid positions. 4,903 positions of the alignment contained gaps in at least one of the taxa. Since the resulting missing data represent less than half of the combined sequence, we included these taxa in the phylogenetic analyses.

Prior to phylogenetic analyses, model selections with different model selection strategies (AIC, AICc, BIC) were performed with ProtTest with all single-gene and multi-gene alignment files. According to the results obtained, we performed maximum-likelihood analysis and 100 maximum-likelihood bootstrap replicates with PHYML applying the WAG+Γ model (gamma shape parameter α = 0.77) of sequence evolution for the concatenated file including 32 loci, and the WAG+I+Γ model (gamma shape parameter α = 1.29, proportion of invariant sites 0.06) for the multi-gene dataset containing 43 gene fragments.

We also applied a mixed-model approach to both datasets using CAT, a previously developed model accounting for site-specific amino acid replacement patterns [44]. To avoid local minimum in tree space search (especially the artefactual attraction of nematodes and platyhelminths, see [45], we used two different starting trees (the most parsimonious one and the one obtained by CAT) and retained the tree with the highest likelihood. Bayesian phylogenetic analyses under the CAT model were performed using the PhyloBayes package (www.lirmm.fr/mab, [46]). For the plain posterior estimation, four independent chains were run for a total number of 15,000 cycles, saving every cycle, and discarding the first 1,500 cycles (burn-in). In all cases, the two independent experiments always lead to the same tree. Therefore, the posterior consensus tree was obtained by pooling both the tree lists of four independent runs. For both models, we measured clade support by non-parametric bootstrap with 100 replicates. To reduce computational burden for the CAT model, a run of 4,000 cycles, discarding the first 1000 as burn-in was performed. The posterior consensus tree was computed for each replicate, and the majority-rule consensus of these 100 trees was our final bootstrap estimate.

In order to test the phylogenetic signal and the contribution of each of the single loci to the general topology, we calculated the number of single-gene trees supporting a given partition of the general topology. To this end, maximum-likelihood trees were constructed from each single-gene alignment with PHYML, applying the model of sequence evolution and the respective parameters according to the model selection with ProtTest. The percentage fraction of single-gene trees containing a particular node is depicted on the branches in Fig. 1. With this approach, and the stringent search criteria in the blast searches, we could exclude the possibility that our alignment files included paralogous genes.

### Hypothesis testing

Alternative topologies constraining monophyly of the Platyhelminthes were compared applying the approximately unbiased (AU) test as implemented in the CONSEL package, using the sidewise likelihood values estimated by PAML. For both datasets, the AU test revealed that the maximum likelihood phylogenies placing *Isodiametra pulchra* as most ancestral taxon, sister group to all remaining bilaterians (Fig. 1, Fig S1), was significantly better based on the available data (*p*>0.01) compared to trees in which the monophyly of the Platyhelminthes was enforced (Table S4).

## Supporting Information

Figure S1 Tree of phylogenetic analysis of 24 species. Phylogenetic analysis of 24 species using partial sequences of 32 genes. The acoel *I. pulchra* appears as a sister group of the rest of the bilaterians, and not as a member of the platyhelminthes. The macrostomorphan *M. lignano* lies basal to other rhabditophoran flatworms (Tricladida, Neodermata). Numbers above nodes refer to the maximum likelihood boostraps. Values below nodes represent bootstrap support under CAT. Circled numbers indicate the percentage of individual-loci trees that supported the respective node in the maximum-likelihood analyses of each data-set separately.
(0.82 MB TIF)

Table S1 Species used for the phylogenetic tree reconstruction.
(0.05 MB DOC)

Table S2 GenBank accession numbers. GenBank accession numbers of the sequences used for the phylogenetic analyses.
(0.19 MB DOC)

Table S3 Length of the sequences used. Length of the sequences used for the phylogenetic analyses.
(0.03 MB DOC)

Table S4 Testing the position of the acoel in the tree. Testing the position of the acoel Isodiametra pulchra in the phylogenetic tree.
(0.04 MB DOC)
