# Supplementary figures and images for: To Be or Not to Be a Flatworm: The Acoel Controversy
Source: PLoS One. 2009 May 11;4(5):e5502. doi: 10.1371/journal.pone.0005502 (PMC2676513; doi:10.1371/journal.pone.0005502)

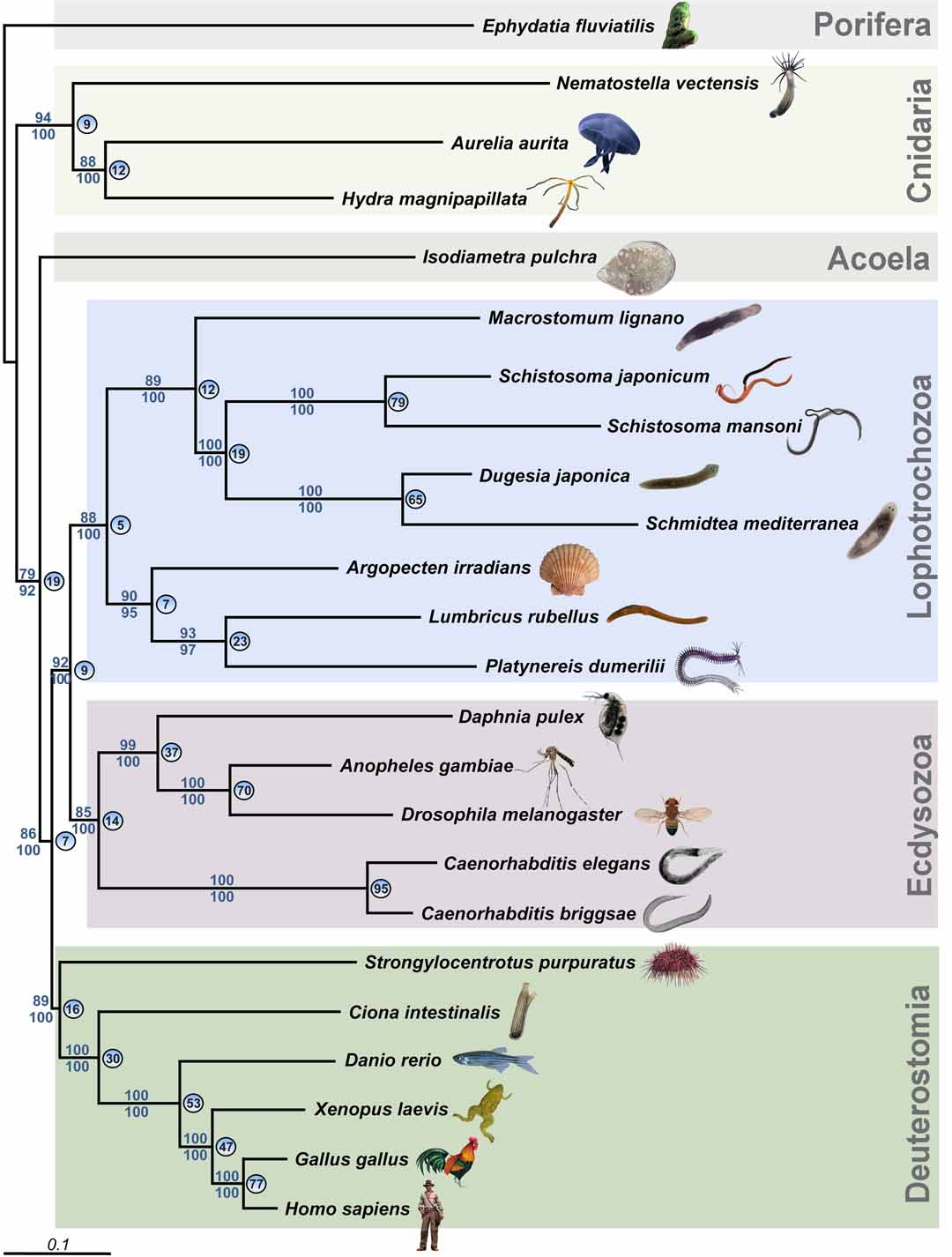

Supplement: Figure S1 — Tree of phylogenetic analysis of 24 species. Phylogenetic analysis of 24 species using partial sequences of 32 genes. The acoel I. pulchra appears as a sister group of the rest of the bilaterians, and not as a member of the platyhelminthes. The macrostomorphan M. lignano lies basal to other rhabditophoran flatworms (Tricladida, Neodermata). Numbers above nodes refer to the maximum likelihood boostraps. Values below nodes represent bootstrap support under CAT. Circled numbers indicate the percentage of individual-loci trees that supported the respective node in the maximum-likelihood analyses of each data-set separately. (0.82 MB TIF) [file pone.0005502.s001.tif]
