# Supplementary material for: To Be or Not to Be a Flatworm: The Acoel Controversy
Source: PLoS One. 2009 May 11;4(5):e5502. doi: 10.1371/journal.pone.0005502 (PMC2676513; doi:10.1371/journal.pone.0005502)
Supplement: Table S1 — Species used for the phylogenetic tree reconstruction. (0.05 MB DOC) [file pone.0005502.s002.doc]

**Table S1 Species used for the phylogenetic tree reconstruction.**

| **Taxon** | **Source** | **Database** | **link** | **version** |
| --- | --- | --- | --- | --- |
| *Argopecten irradians* | EST | NCBI GenBank | www.ncbi.nih.gov | 05/2005 |
| *Anopheles gambiae* | Genome | ENSEMBL | www.ensembl.org | MOZ2 |
| *Aurelia aurita* | EST | CDB, RIKEN Kobe | www.cdb.riken.jp | 03/2005 |
| *Caenorhabditis briggsae* | Genome | Sanger Institute | www.sanger.ac.uk | cb25.agp8 |
| *Caenorhabditis elegans* | Genome | ENSEMBL | www.ensembl.org | WS140 |
| *Ciona intestinalis* | Genome | JGI | genome.jgi-psf.org | V 2.0 |
| *Danio rerio* | Genome | ENSEMBL | www.ensembl.org | WTSI Zv5 |
| *Daphnia pulex* | Genome | wFleaBase | wfleabase.org | 07/2005 |
| *Drosophila melanogaster* | Genome | ENSEMBL | www.ensembl.org | BGDP 4 |
| *Dugesia japonica* | EST | NCBI GenBank | www.ncbi.nih.gov | 05/2005 |
| *Ephydatia fluviatilis* | EST | CDB, RIKEN Kobe | www.cdb.riken.jp | 03/2005 |
| *Gallus gallus* | Genome | ENSEMBL | www.ensembl.org | WASHUC1 |
| *Homo sapiens* | Genome | NCBI GenBank | www.ncbi.nih.gov | NCBI 35 |
| *Hydra magnipapillata* | EST | NCBI GenBank | www.ncbi.nih.gov | 05/2005 |
| *Isodiametra pulchra* | EST | Institute of Zoology, Innsbruck | zoology.uibk.ac.at | 01/2005 |
| *Lumbricus rubellus* | EST | NCBI GenBank | www.ncbi.nih.gov | 05/2005 |
| *Macrostomum lignano* | EST | Institute of Zoology, Innsbruck | zoology.uibk.ac.at | 01/2005 |
| *Nematostella vectensis* | EST | Sars | www.sars.no | 02/2005 |
| *Platynereis dumerilii* | EST | EMBL | www.embl.org | 03/2005 |
| *Schistosoma japonicum* | Genome | NCBI GenBank | www.ncbi.nih.gov | 05/2005 |
| *Schistosoma mansoni* | Genome | Sanger Institute | www.sanger.ac.uk | 05/2005 |
| *Schmidtea mediterranea* | EST | NCBI GenBank | www.ncbi.nih.gov | 05/2005 |
| *Strongylocentrotus purpuratus* | EST | NCBI GenBank | www.ncbi.nih.gov | 05/2005 |
| *Xenopus laevis* | EST | NCBI GenBank | www.ncbi.nih.gov | 05/2005 |
