# Supplementary material for: To Be or Not to Be a Flatworm: The Acoel Controversy
Source: PLoS One. 2009 May 11;4(5):e5502. doi: 10.1371/journal.pone.0005502 (PMC2676513; doi:10.1371/journal.pone.0005502)
Supplement: Table S2 — GenBank accession numbers. GenBank accession numbers of the sequences used for the phylogenetic analyses. (0.19 MB DOC) [file pone.0005502.s003.doc]

**Table S2 GenBank accession numbers of the sequences used for the phylogenetic analyses.**

| Annotation according to Human Genome | *Argopecten irradians* | *Anopheles gambiae* | *Aurelia aurita* | *Caenorhabditis briggsae* | *Caenorhabditis elegans* | *Ciona intestinalis* | *Danio rerio* | *Daphnia pulex* |
| --- | --- | --- | --- | --- | --- | --- | --- | --- |
| ARF (ADP-Ribosylation Factor related)-Like (arf-5) | CB413913 | XP_312971 | BY999811 | CBP00452 | Cel#S5748900 | ci0100134949 | DARP00000041514 | WFes0005985 |
| dynein light chain | CN783017 | XP_321810 |  | CBP04582 | Cel#S20159347 | ci0100136300 | DARP00000015065 | WFes0005161 |
| protein YR-29 | CB414180 | EAA43915 | BY999822 | CBP04643 | Cel#S5828621 | ci0100131031 | DARP00000003036 | WFes0011810 |
| suppressor of presenilin defect SPR-2/SET family | CB415884 | XP_313356 |  | CBP05129 | Cel#S5770043 | ci0100142619 | DARP00000040740 | WFes0009589 |
| histone deacetylase (hda-3) | AF526222 | EAA01056 | BY999813 | CBP06924 | Cel#S5833045 | ci0100146906 | DARP00000025705 | WFes0001654 |
| ubiquitin-conjugating enzyme effete (eff) | CV660846 | EAA06420 | BY999838 | CBP15507 | Cel#S5699559 | ci0100137855 | DARP00000049284 | WFes0004602 |
| stomatin (sto-1) | CB412871 | XP_314315 |  | CBP09746 | Cel#S5708768 | ci0100145817 | DARP00000050518 | WFes0004579 |
| GTP-binding protein rab-2 | CK484440 | XP_315402 | BY999812 | CBP02928 | Cel#S5713747 | ci0100134294 | DARP00000003571 | WFes0010796 |
| sarco-endoplasmic reticulum Calcium ATPase (sca-1) | CN782368 | XP_316251 | BY999828 | CBP04306 | Cel#S5737339 | ci0100154411 | DARP00000047200 | WFes0011544 |
| transmembrane protein TM9SF3 | CB414852 | XP_314301 | BY999817 | CBP02050 | Cel#S5764279 | ci0100141453 | DARP00000024049 | WFes0010664 |
| GTP-binding protein rab-21 | CB413124 | EAA43937 | BY999821 | CBP00310 | Cel#S5801025 | ci0100133089 | DARP00000033828 | WFes0008612 |
| glutathione peroxidase (PHGPx) | CB415992 | EAA44749 | BY999845 | CBP14919 | Cel#S18393054 | ci0100131438 | DARP00000008425 | WFes0010181 |
| ribosomal protein, large subunit (rpl-5) | CN783365 | EAL39026 | BY999847 | CBP20815 | Cel#S5808157 | ci0100131973 | DARP00000007676 | WFes0002644 |
| nascent polypetide-associated complex alpha chain | CN783000 | EAA04708 | BY999826 | CBP06992 | Cel#S20200406 | ci0100133497 | DARP00000025953 | WFes0000922 |
| transformer-2 sex-determining protein (tra-2) | CN783011 | XP_318685 | BY999815 | CBP09289 | Cel#S5734196 | ci0100151685 | DARP00000010426 | WFes0003280 |
| cyclophilin-1 (cyp-1) | CB416699 | EAA06299 | BY999832 | CBP07574 | Cel#S5859584 | ci0100130790 | DARP00000003641 | WFes0010488 |
| Ser/Thr protein phosphatase (pph-4.1) | CK484164 | XP_310323 | BY999841 | CBP02888 | Cel#S5848828 | ci0100130621 | DARP00000045533 | WFes0005120 |
| development. regulated GTP binding protein 1 like (drg-1) | CB415403 | XP_310117 | BY999818 | CBP03236 | Cel#S5731359 | ci0100136598 | DARP00000026443 | WFes0002156 |
| clathrin coat assembly protein (ap-2) | CF197494 | EAA04682 | BY999820 | CBP07995 | Cel#S5571117 | ci0100140541 | DARP00000003505 | WFes0012344 |
| NADH dehydrogenase | CB414196 | EAA03768 | BY999835 | CBP17132 | Cel#S5712850 | ci0100137610 | DARP00000004700 | WFes0007165 |
| G Protein, Beta subunit (gbp-1) | CF197666 | XP_315941 | BY999824 | CBP00625 | Cel#S5806578 | ci0100145646 | DARP00000018443 | WFes0003380 |
| cysteine protease related (cpr-6) | CK484454 | XP_313835 | BY999831 | CBP02635 | Cel#S6657456 | ci0100139601 | DARP00000022790 | WFes0010856 |
| neural RNA-binding protein MSI-1 | CN783009 | XP_321067 | BY999825 | CBP00957 | Cel#S5854125 | ci0100141381 | DARP00000049341 | WFes0011990 |
| isocitrate dehydrogenase 3 alpha | AY551096 | EAA08136 | BY999843 | CBP22694 | Cel#S5838537 | ci0100132137 | DARP00000039951 | WFes0008271 |
| phosphate carrier protein | CN782763 | XP_313339 | BY999833 | CBP01631 | Cel#S5782584 | ci0100142793 | DARP00000028689 | WFes0000754 |
| fructose-1,6-bisphosphate aldolase class-I | CF197725 | EAA08079 | BY999814 | CBP03719 | Cel#S6657599 | ci0100140090 | DARP00000010608 | WFes0002857 |
| DNaJ domain - prokaryotic heat shock protein (dnj-12) | CB413611 | XP_316024 | BY999830 | CBP08309 | Cel#S5838712 | ci0100134317 | DARP00000010419 | WFes0009941 |
| propionyl-CoA carboxylase | CK484530 | XP_319416 |  | CBP18815 | Cel#S5709843 | ci0100130281 | DARP00000020266 | WFes0012366 |
| glyceraldehyde-3-phosphate dehydrogenase 3 (g3p3) | CF197648 | XP_318655 | BY999840 | CBP05911 | Cel#S5709307 | ci0100132109 | DARP00000009462 | WFes0000815 |
| enolase | CB412647 | EAA12254 | BY999827 | CBP00835 | Cel#S5798500 | ci0100151116 | DARP00000002923 | WFes0012352 |
| guanine nucleotide-binding protein (gsa-1) | CK484293 | EAA13872 | BY999816 | CBP00888 | Cel#S5774238 | ci0100139933 | DARP00000021134 | WFes0008664 |
| ADP-ribosylation factor related (arf-3) | CB415168 | EAA00461 | BY999836 | CBP04142 | Cel#S18388668 | ci0100131662 | DARP00000015805 | WFes0002180 |
| stress-induced-phosphoprotein 1 (stip1) | CN782933 | XP_319365 | BY999819 | CBP06756 | Cel#S5711988 | ci0100134828 | DARP00000041440 | WFes0004936 |
| ATP synthase subunit (atp-2) | CB414069 | XP_320423 | BY999810 | CBP11745 | Cel#S5745340 | ci0100141566 | DARP00000031487 | WFes0009730 |
| importin alpha (ima-3) | CK484177 | EAA01688 | BY999823 | CBP07179 | Cel#S5779722 | ci0100145134 | DARP00000000968 | WFes0012354 |
| malate dehydrogenase (mdh-1) | CN783142 | XP_321163 | BY999839 | CBP03693 | Cel#S5736548 | ci0100147369 | DARP00000048494 | WFes0003656 |
| methionine adenosyltransferase (minute 2) | CK484446 | XP_307863 | BY999829 | CBP04704 | Cel#S5788524 | ci0100137824 | DARP00000011513 | WFes0006324 |
| cathepsin L (cpl-1) | CK484442 | XP_320687 | BY999834 | CBP05395 | Cel#S5711615 | ci0100137495 | DARP00000039710 | WFes0008230 |
| citrate synthase | CB415675 | EAA00454 | BY999844 | CBP02471 | Cel#S5732335 | ci0100141577 | DARP00000031758 | WFes0004488 |
| pyruvate kinase | CV548400 | EAA10555 |  | CBP04065 | Cel#S5758294 | ci0100150597 | DARP00000017190 | WFes0004001 |
| elongation factor 1 (ef-1 beta) | CN783023 | XP_314575 | BY999842 | CBP12290 | Cel#S5645562 | ci0100152007 | DARP00000018258 | WFes0004229 |
| heat shock protein, abnormal Dauer Formation (daf-21) | CN782963 | EAA04712 | BY999846 | CBP01204 | Cel#S5713933 | ci0100134890 | DARP00000022302 | WFes0012030 |
| ATP synthase mitochondrial | CB416006 | EAA12854 | BY999848 | CBP12184 | Cel#S5760730 | ci0100150565 | DARP00000007943 | WFes0000286 |

| Annotation according to Human Genome | *Drosophila melanogaster* | *Dugesia japonica* | *Ephydatia fluviatilis* | *Gallus gallus* | *Homo sapiens* | *Hydra magnipapillata* | *Isodiametra pulchra* | *Lumbricus rubellus* |
| --- | --- | --- | --- | --- | --- | --- | --- | --- |
| ARF (ADP-Ribosylation Factor related)-Like (arf-5) | Dm#S13547177 | BP191288 | BY999850 | XP_421589 | NP_064535 | Hma#S19803132 | GH985273 | CO048228 |
| dynein light chain | Dm#S13275431 | BP189019 | BY999857 | XP_415908 | NP_542408 | Hma#S19797201 | GH985274 | CV462247 |
| protein YR-29 | Dm#S13548579 | BP188659 | BY999872 | XP_424793 | NP_055701 | Hma#S21730524 | GH985275 | DR697017 |
| suppressor of presenilin defect SPR-2/SET family | Dm#S13273778 | BP187634 | BY999886 | XP_415493 | NP_003002 | Hma#S21940069 | GH985276 | CF426664 |
| histone deacetylase (hda-3) | Dm#S13274380 | BP187805 | BY999853 | AAB96925 | NP_003874 | Hma#S22254563 | GH985277 | CF415930 |
| ubiquitin-conjugating enzyme effete (eff) | Dm#S13560503 | BP189372 | BY999856 | CAG31534 | NP_003331 | Hma#S19838697 | GH985278 | CF810354 |
| stomatin (sto-1) | Dm#S13276461 | BP189744 | BY999878 | XP_425632 | NP_660329 | Hma#S19807550 | GH985279 | CF810072 |
| GTP-binding protein rab-2 | Dm#S13279881 | BP191274 | BY999884 | 2209256A | NP_002856 | Hma#S21739576 | GH985280 | CO048070 |
| sarco-endoplasmic reticulum Calcium ATPase (sca-1) | Dm#S13554756 | BP190880 | BY999865 | B40812 | NP_733765 | Hma#S21743907 | GH985281 | CO048347 |
| transmembrane protein TM9SF3 | Dm#S13545825 | BP186162 | BY999851 | XP_421629 | NP_064508 | Hma#S20102930 | GH985282 | CO408534 |
| GTP-binding protein rab-21 | Dm#S13565354 | BP188881 | BY999889 | CAG32679 | NP_958842 | Hma#S21741767 | GH985283 | CA036010 |
| glutathione peroxidase (PHGPx) | Dm#S13546644 | BP185217 | BY999858 | AAM18080 | NP_002076 | Hma#S22416730 | GH985284 | CO047918 |
| ribosomal protein, large subunit (rpl-5) | Dm#S13282588 | BP185347 | BY999885 | BAA01581 | NP_000960 | Hma#S21731253 | GH985285 | CF809901 |
| nascent polypetide-associated complex alpha chain | Dm#S13279945 | BP188417 | BY999864 | XP_418516 | NP_005585 | Hma#S22417176 | GH985286 | CF416630 |
| transformer-2 sex-determining protein (tra-2) | Dm#S13279424 | BP186828 | BY999854 | AAG35783 | NP_004584 | Hma#S22252700 | GH985287 | DR696987 |
| cyclophilin-1 (cyp-1) | Dm#S13285541 | BP187211 | BY999860 | XP_421600 | NP_005720 | Hma#S19838153 | GH985288 | BF422612 |
| Ser/Thr protein phosphatase (pph-4.1) | Dm#S13561758 | BP185501 | BY999870 | BAA04481 | NP_002711 | Hma#S22324816 | GH985289 | CF416397 |
| development. regulated GTP binding protein 1 like (drg-1) | Dm#S13283850 | BP185110 | BY999887 | XP_415255 | NP_004138 | Hma#S20114425 | GH985290 | CO046568 |
| clathrin coat assembly protein (ap-2) | Dm#S13282866 | BP189454 | BY999852 | CAG32151 | NP_001275 | Hma#S19838988 | GH985291 | CO048105 |
| NADH dehydrogenase | Dm#S13281030 | BP186871 | BY999875 | XP_424129 | NP_066552 | Hma#S22417069 | GH985292 | DR008232 |
| G Protein, Beta subunit (gbp-1) | Dm#S19187045 | BP188362 | BY999882 | XP_417564 | NP_002065 | Hma#S19834010 | GH985293 | DR008257 |
| cysteine protease related (cpr-6) | Dm#S13280855 | BP190448 | BY999868 | XP_429301 | NP_001899 | Hma#S21739414 | GH985294 | CF611247 |
| neural RNA-binding protein MSI-1 | Dm#S13559305 | BP190178 |  | XP_415912 | NP_002433 | Hma#S22417046 | GH985295 | CF799251 |
| isocitrate dehydrogenase 3 alpha | Dm#S13561836 | BP186234 | BY999863 | XP_413748 | NP_005521 | Hma#S22416675 | GH985296 | CF416299 |
| phosphate carrier protein | Dm#S13548830 | BP188544 | BY999871 | XP_416165 | NP_998776 | Hma#S21744342 | GH985297 | CO047799 |
| fructose-1,6-bisphosphate aldolase class-I | Dm#S13549033 | BP188363 | BY999869 | 1609082A | NP_005156 | Hma#S21743493 | GH985298 | CO058459 |
| DNaJ domain - prokaryotic heat shock protein (dnj-12) | Dm#S13560639 | BP189910 | BY999890 | XP_414110 | NP_005871 | Hma#S22253110 | GH985299 | CF839048 |
| propionyl-CoA carboxylase | Dm#S16606762 | BP188661 | BY999874 | XP_416970 | NP_000273 | Hma#S22251899 | GH985300 | CO058316 |
| glyceraldehyde-3-phosphate dehydrogenase 3 (g3p3) | Dm#S13544729 | BP188880 | BY999879 | AAD02474 | NP_002037 | Hma#S21741253 | GH985301 | DR076781 |
| enolase | Dm#S20791170 | BP185870 | BY999876 | BAA07132 | NP_001419 | Hma#S22160364 | GH985302 | CF810393 |
| guanine nucleotide-binding protein (gsa-1) | Dm#S13282232 | BP190269 | BY999873 | AAA50559 | NP_006089 | Hma#S21738509 | GH985303 | CF416772 |
| ADP-ribosylation factor related (arf-3) | Dm#S13280152 | BP186060 | BY999866 | XP_418504 | NP_001650 | Hma#S21740581 | GH985304 | CO046736 |
| stress-induced-phosphoprotein 1 (stip1) | Dm#S13280018 | BP189253 | BY999849 | CAG32677 | NP_006810 | Hma#S19805149 | GH985305 | CO046521 |
| ATP synthase subunit (atp-2) | Dm#S13563416 | BP185647 | BY999881 | CAG31468 | NP_001677 | Hma#S21743864 | GH985306 | CO058503 |
| importin alpha (ima-3) | Dm#S13561056 | BP185681 | BY999867 | XP_422815 | NP_002259 | Hma#S21730850 | GH985307 | CF416241 |
| malate dehydrogenase (mdh-1) | Dm#S13547688 | BP187480 | BY999883 | XP_41576 | NP_005909 | Hma#S21741565 | GH985308 | CF809730 |
| methionine adenosyltransferase (minute 2) | Dm#S13565549 | BP185234 | BY999861 | XP_421512 | NP_000420 | Hma#S21742119 | GH985309 | DR696910 |
| cathepsin L (cpl-1) | Dm#S13558037 | BP186259 | BY999880 | XP_425038 | NP_001903 | Hma#S22416385 | GH985310 | CO048067 |
| citrate synthase | Dm#S13562972 | BP188499 | BY999859 | 1CSH | NP_004068 | Hma#S21740726 | GH985311 | CO869418 |
| pyruvate kinase | Dm#S13565734 | BP187286 | BY999855 | AAA49020 | NP_002645 | Hma#S21742413 | GH985312 | DR008999 |
| elongation factor 1 (ef-1 beta) | Dm#S13283625 | BP189189 | BY999888 | AAD16874 | NP_001950 | Hma#S19835007 | GH985313 | CF809688 |
| heat shock protein, abnormal Dauer Formation (daf-21) | Dm#S13548802 | BP186894 | BY999877 | CAA49704 | NP_031381 | Hma#S21730455 | GH985314 | CO046643 |
| ATP synthase mitochondrial | Dm#S13545263 | BP185436 | BY999862 | XP_417296 | NP_005165 | Hma#S22416387 | GH985315 | CO046723 |

| Annotation according to Human Genome | *Macrostomum lignano* | *Nematostella vectensis* | *Platynereis dumerilii* | *Schistosoma japonicum* | *Schistosoma mansoni* | *Schmidtea mediterranea* | *Strongylocentrotus purpuratus* | *Xenopus laevis* |
| --- | --- | --- | --- | --- | --- | --- | --- | --- |
| ARF (ADP-Ribosylation Factor related)-Like (arf-5) | EG951878 | FC286018 | EZ114341 | CV750616 | c001431653 | AY068214 | CD307210 | AAH81079 |
| dynein light chain | EG957146 | FC253008 | EZ114342 | AW061368 | c002626719 | AY066082 | CD340237 | AAH57215 |
| protein YR-29 | EG956962 | FC241709 |  | BU802504 | c002521284 | AY067753 | CD289545 | AAH73255 |
| suppressor of presenilin defect SPR-2/SET family | EG956950 | FC255577 | EZ114343 | CV754402 | c002521763 | AY068217 | CD304907 | BAA84766 |
| histone deacetylase (hda-3) | EG957403 | FC209803 | EZ114344 | CV739039 | c001329131 | AY066136 | CD290555 | AAH70873 |
| ubiquitin-conjugating enzyme effete (eff) | EG955244 | FC294134 | EZ114345 | BU716248 | c002521770 | AY066205 | CD304598 | BAD06215 |
| stomatin (sto-1) | EG954870 | FC298702 | GO599622 | BU773040 | c001434190 | AY067502 | CD310180 | AAH54307 |
| GTP-binding protein rab-2 | EG952567 | FC298007 | GO599623 | BU798155 | c000622399 | AY067067 | CD321498 | AAH71068 |
| sarco-endoplasmic reticulum Calcium ATPase (sca-1) | EG953277 | FC291758 | EZ114346 | BU801481 | c000120762 | AY068245 | CD295387 | AAH44063 |
| transmembrane protein TM9SF3 | EG953165 | FC284469 | GO599624 | BU799522 | c000224652 | AY066103 | CD306288 | AAH60487 |
| GTP-binding protein rab-21 | EG951489 | FC275517 | GO599625 | AY223153 | c002216523 | AY067001 | CD295683 | AAH43866 |
| glutathione peroxidase (PHGPx) | EG955284 | FC235418 | EZ114347 | BU794956 | c000221860 | AY067488 | CD308731 | AAH84801 |
| ribosomal protein, large subunit (rpl-5) | EG957335 | FC243177 | EZ114348 | AY223167 | c002219290 | AY067191 | CD295854 | AAH42258 |
| nascent polypetide-associated complex alpha chain | EG957393 | FC237683 | EZ114349 | BU792163 | c001030066 | AY067319 | CD312106 | AAH72044 |
| transformer-2 sex-determining protein (tra-2) | EG953761 | FC264770 | GO599626 | BU803183 | c001537224 | AY067222 | CD311018 | AAH72952 |
| cyclophilin-1 (cyp-1) | EG955246 | FC204893 | GO599627 | BU711047 | c001435706 | AY067044 | CD323775 | AAH41536 |
| Ser/Thr protein phosphatase (pph-4.1) | EG952254 | FC293126 | GO599628 | BU714685 | c002522523 | AY066113 | CD290875 | AAH72026 |
| development. regulated GTP binding protein 1 like (drg-1) | EG957270 | FC266289 | EZ114350 | BU766819 | c001632897 | AY066984 | CD295686 | I51426 |
| clathrin coat assembly protein (ap-2) |  | FC292813 | GO599629 | BU793733 | c002053621 | AY067778 | CD331934 | AAH41251 |
| NADH dehydrogenase |  | DV088230 |  | BU724416 | c002431081 | AY067472 | CD316741 | AAH45095 |
| G Protein, Beta subunit (gbp-1) |  | FC249273 | GO599630 | CV741688 | c000422644 | AY067666 | CD308608 | AAH84263 |
| cysteine protease related (cpr-6) | EG954743 | FC305086 | GO599631 | AY226984 | c001535173 | AY067658 | CD290018 | AAH46667 |
| neural RNA-binding protein MSI-1 | EG957671 | FC259212 | EZ114351 | BU714849 | c000924413 | AY068302 | CD338272 | AAA50004 |
| isocitrate dehydrogenase 3 alpha | EG955494 | FC315751 | GO599635 | BU717274 | c000325544 | AY068135 | CD295425 | AAH73655 |
| phosphate carrier protein | EG957299 | FC259321 | EZ114363 | BU725811 | c002722173 | AY066609 | CD317295 | AAH86148 |
| fructose-1,6-bisphosphate aldolase class-I | EG955205 | FC234422 | GO599636 | BU719213 | c002630979 | AY068106 | CD336746 | AAB31152 |
| DNaJ domain - prokaryotic heat shock protein (dnj-12) | EG953337 | FC236643 | GO599637 | CV740057 | c000826521 | AY068379 | CD310765 | AAH46954 |
| propionyl-CoA carboxylase |  | FC287135 | GO599633 | CV745907 | c001737995 | AY067470 | CD320478 | AAH74151 |
| glyceraldehyde-3-phosphate dehydrogenase 3 (g3p3) |  | FC280134 | GO599632 | U75511 | c000010195 | AY068133 | CD308722 | AAH43972 |
| enolase |  | FC251458 |  | L23324 | c000019895 | AY068130 | CD294632 | AAH54169 |
| guanine nucleotide-binding protein (gsa-1) | EG955934 | FC252778 | EZ114356 | BU802517 | c000826311 | AY067181 | CD304923 | AAH86231 |
| ADP-ribosylation factor related (arf-3) | EG957160 | FC298266 | EZ114357 | BU803433 | c002433078 | AY067452 | CD294713 | AAH42337 |
| stress-induced-phosphoprotein 1 (stip1) | EG956819 | FC214501 | GO599634 | BU803102 | c001941908 | AY067280 | CD309676 | AAH46709 |
| ATP synthase subunit (atp-2) | EG957097 | FC267370 | EZ114353 | BU724775 | c000925643 | AY067818 | CD293070 | AAH46741 |
| importin alpha (ima-3) | EG957256 | FC270669 | EZ114358 | BU718952 | c002142791 | AY068344 | CD320293 | AAH70983 |
| malate dehydrogenase (mdh-1) |  | FC254662 | EZ114359 | CV747493 | c001435661 | AY068134 | CD289594 | AAH87199 |
| methionine adenosyltransferase (minute 2) | EG957854 | FC264739 | EZ114354 | BU802473 | c000121632 | AY067803 | CD308648 | AAH86018 |
| cathepsin L (cpl-1) |  | FC275062 | EZ114360 | U38476 | c000924518 | AY067853 | CD293077 | AAH80004 |
| citrate synthase |  | FC281725 | EZ114355 | AY223083 | c000821592 | AY068745 | CD310796 | AAH46571 |
| pyruvate kinase |  | FC287146 |  | BU803210 | c000222561 | AY068121 | CD296879 | AAA63581 |
| elongation factor 1 (ef-1 beta) | EG953283 | FC305688 | EZ114361 | BU803638 | c002521772 | AY067677 | CD307350 | AAH82168 |
| heat shock protein, abnormal Dauer Formation (daf-21) |  | FC293868 | EZ114352 | AY223382 | c002431523 | AY067271 | AI478066 | AAH87184 |
| ATP synthase mitochondrial | EG950383 | FC239408 | EZ114362 | BU796243 | c000724135 | AY067816 | CD309598 | AAH72367 |
