# Supplementary material for: To Be or Not to Be a Flatworm: The Acoel Controversy
Source: PLoS One. 2009 May 11;4(5):e5502. doi: 10.1371/journal.pone.0005502 (PMC2676513; doi:10.1371/journal.pone.0005502)
Supplement: Table S3 — Length of the sequences used. Length of the sequences used for the phylogenetic analyses. (0.03 MB DOC) [file pone.0005502.s004.doc]

Table S3: Length of the sequences used for the phylogenetic analyses.

| ID *isodiametra pulchra* | Annotation according to Human Genome | EST length (bp) | Amino acids used |
| --- | --- | --- | --- |
| Cpu_aW_001_A11* | ARF (ADP-Ribosylation Factor related)-Like (arf-5) | 909 | 185 |
| Cpu_aW_008_G22* | dynein light chain | 876 | 89 |
| Cpu_aW_008_I10* | protein YR-29 | 1004 | 173 |
| Cpu_aW_010_F20* | suppressor of presenilin defect SPR-2/SET family | 812 | 124 |
| Cpu_aW_012_L13* | histone deacetylase (hda-3) | 862 | 269 |
| Cpu_aW_015_L19* | ubiquitin-conjugating enzyme effete (eff) | 730 | 147 |
| Cpu_aW_016_F24* | stomatin (sto-1) | 783 | 226 |
| Cpu_aW_016_M12* | GTP-binding protein rab-2 | 831 | 162 |
| Cpu_aW_016_N17* | sarco-endoplasmic reticulum Calcium ATPase (sca-1) | 789 | 263 |
| Cpu_aW_017_P11* | transmembrane protein TM9SF3 | 779 | 203 |
| Cpu_aW_019_I02* | GTP-binding protein rab-21 | 803 | 153 |
| Cpu_aW_019_L09* | glutathione peroxidase (PHGPx) | 701 | 140 |
| Cpu_aW_020_P17* | ribosomal protein, large subunit (rpl-5) | 853 | 204 |
| Cpu_aW_021_G05* | nascent polypetide-associated complex alpha chain | 800 | 109 |
| Cpu_aW_022_P03* | transformer-2 sex-determining protein (tra-2) | 879 | 117 |
| Cpu_aW_024_D23* | cyclophilin-1 (cyp-1) | 879 | 165 |
| Cpu_aW_025_B24* | Ser/Thr protein phosphatase (pph-4.1) | 732 | 239 |
| Cpu_aW_025_L20* | developmentally regulated GTP binding protein 1 (drg-1) | 961 | 280 |
| Cpu_aW_026_C20 | clathrin coat assembly protein (ap-2) | 855 | 182 |
| Cpu_aW_026_K01 | NADH dehydrogenase | 781 | 208 |
| Cpu_aW_027_M24 | G Protein, Beta subunit (gbp-1) | 861 | 264 |
| Cpu_aW_029_J24* | cysteine protease related (cpr-6) | 857 | 239 |
| Cpu_aW_030_C06* | neural RNA-binding protein MSI-1 | 907 | 110 |
| Cpu_aW_CL1191 * | isocitrate dehydrogenase 3 alpha | 933 | 241 |
| Cpu_aW_CL1439* | phosphate carrier protein | 1137 | 277 |
| Cpu_aW_CL1441 * | fructose-1,6-bisphosphate aldolase class-I | 893 | 285 |
| Cpu_aW_CL1488 * | DNaJ domain - prokaryotic heat shock protein (dnj-12) | 1275 | 227 |
| Cpu_aW_CCL166 | propionyl-CoA carboxylase | 2281 | 444 |
| Cpu_aW_CL20 * | glyceraldehyde-3-phosphate dehydrogenase 3 (g3p3) | 1233 | 334 |
| Cpu_aW_CL24 | enolase | 1550 | 412 |
| Cpu_aW_CL282 * | guanine nucleotide-binding protein (gsa-1) | 880 | 276 |
| Cpu_aW_CL320 * | ADP-ribosylation factor related (arf-3) | 1026 | 179 |
| Cpu_aW_CL358 * | stress-induced-phosphoprotein 1 (stip1) | 1160 | 147 |
| Cpu_aW_CL36 * | ATP synthase subunit (atp-2) | 1645 | 466 |
| Cpu_aW_CL415 * | importin alpha (ima-3) | 1199 | 198 |
| Cpu_aW_CL582 | malate dehydrogenase (mdh-1) | 872 | 259 |
| Cpu_aW_CL60 * | methionine adenosyltransferase (minute 2) | 1252 | 378 |
| Cpu_aW_CL638 | cathepsin L (cpl-1) | 994 | 172 |
| Cpu_aW_CL65 | citrate synthase | 1497 | 427 |
| Cpu_aW_CL667 | pyruvate kinase | 1493 | 386 |
| Cpu_aW_CL678 * | elongation factor 1 (ef-1 beta) | 1026 | 117 |
| Cpu_aW_CL7 | heat shock protein, abnormal Dauer Formation (daf-21) | 2388 | 524 |
| Cpu_aW_CL981 * | ATP synthase mitochondrial | 835 | 218 |

an * indicates the loci used in the small dataset
