# Supplementary material for: To Be or Not to Be a Flatworm: The Acoel Controversy
Source: PLoS One. 2009 May 11;4(5):e5502. doi: 10.1371/journal.pone.0005502 (PMC2676513; doi:10.1371/journal.pone.0005502)
Supplement: Table S4 — Testing the position of the acoel in the tree. Testing the position of the acoel Isodiametra pulchra in the phylogenetic tree. (0.04 MB DOC) [file pone.0005502.s005.doc]

**Table S4 Testing the position of the acoel Isodiametra pulchra in the phylogenetic tree.**

| **Position of *Isodiametra pulchra*** | **loglk** | **Δloglk** | ***P* approx. unbiased test** |
| --- | --- | --- | --- |
| Dataset including *Macrostomum lignano* | |  |  |
| Basal bilaterian | - 215836.0 | 0.0 |  |
| Basal to Rhabditophora | - 215953.2 | -117.2 | 0.008 |
| Basal to Tricladida and Neodermata | - 215979.9 | -143.9 | 0.008 |
|  | |  |  |
| Dataset without *Macrostomum lignano* | |  |  |
| Basal bilaterian | - 205935,1 | 0.0 |  |
| Basal to Tricladida and Neodermata | - 206038.9 | -102,9 | 0.002 |

According to the approximately unbiased (AU) test as implemented in the CONSEL package (using the sidewise likelihood values estimated by PAML), the phylogeny placing *Isodiametra pulchra* as most ancestral taxon and sister group to all remaining bilaterians is significantly better than the enforced monophyly of the Platyhelminthes.
